# Supplementary material for: The Psychosocial Burden of Families with Childhood Blood Cancer
Source: Int J Environ Res Public Health. 2022 Jan 5;19(1):599. doi: 10.3390/ijerph19010599 (PMC8744617; doi:10.3390/ijerph19010599)
Supplement: Supplementary file 1 [file ijerph-19-00599-s001.zip › ijerph-1465120-supplementary.pdf]

Supplementary Material for:

The Psychosocial Burden of Families with Childhood Blood Cancer

by Florencia Borrescio-Higa and Nieves Valdés

Supplementary Material A.

Representativeness of the sample

To assess the representativeness of the sample, we compare descriptive statistics with those obtained from the "First Report of the Chilean National Childhood Cancer Registry" (RENCI, from the Spanish acronym), that collected information on children aged 14 years old or less, diagnosed with any type of cancer between 2007 and 2011 [1]. The RENCI Report includes 2,404 cases, with an annual average of 480 new diagnosis. Note that the comparison is not precise, since the time spam of the year of diagnosis covered in the RENCI Report is older than in the survey. However, it is the unique source of information available for the country. Moreover, the RENCI Report includes information aggregated for children with any type of cancer, while our study is focused on blood cancer.

The descriptive statistics reported in Table 2 show that our sample is very similar to the children registered in RENCI in the distribution of ages at diagnosis, and the type of blood cancer. The proportion of children residing in the Metropolitan region by the time of the diagnosis is higher in the sample surveyed in our study, has a higher proportion of girls, and a higher proportion of children covered by a private health insurance, than the population of the RENCI Report. The last finding suggests that our dataset includes a higher proportion of children with higher SES than the Chilean population. Nevertheless, Borrescio-Higa and Valdes (2021) show that in 2018, 42% of hospitalized pediatric patients who face some kind of copayment were covered by a private health insurance [2].

Table S1. Descriptive statistics: Sample vs. the First Report of the Chilean National Childhood Cancer Registry.

	Working sample		RENCI 2007-2011	
Variable	Number	Percent	Number	Percent
Child's information				
Gender				
Female	46	53.5%	1068	44.4%
Male	40	46.5%	1336	55.6%
Age at diagnosis (years)				
≤ 4	40	46.5%	1043	43.4%
5-9	30	34.9%	650	27.0%
10-14	16	18.6%	711	29.6%
Region of treatment [(a)]				
Metropolitan region	58	67.4%	1260	52.4%
Other	28	32.6%	1144	47.6%
Diagnosis				
Leukemia	79	92%	965	93%
Non-Hodgkin Lymphoma	7	8%	74	7%
Health Insurance				
Private	39	45.3%	131	10.4%
Public	43	50.0%	1998	83.1%
Other	4	4.7%	74	6.5%

Note: [(a)] Missing if child is dead at the time of the survey.

References

1. Ministerio de Salud *Primer informe del registro nacional de cáncer infantil de Chile (menores de 15 años), RENCI*; 2018;
2. Borrescio-Higa, F.; Valdés, N. Medical Cost of Cancer Care for Privately Insured Children in Chile. *International Journal of Environmental Research and Public Health* **2021**, *18*, 6746, doi:10.3390/ijerph18136746.

Supplementary Material B: Survey questions on the impact of the childhood cancer used in the analyses.

- Which of the following activities has [care giver] done? Alternatives:
1. Takes child to control
2. Takes child to treatment
3. Looks for financial aid
4. Accompanies child during hospital stay
5. Home modifications
6. Aid with rehabilitation
7. Help with school activities/child's education
8. Other [Specify]

- Related to the work status of [care giver], which of the following has happened as a consequence of the illness of [patient's name] ? Alternatives:
1. Increased housework
2. Increased workload
3. Job absenteeism
4. Job layoff
5. Quit job
6. Change job
7. Reduction in salary
8. None of the above
9. Other
10. Does not know

- Did [patient's name] dropped out of school as a consequence of the illness? Response: Yes-No

- What kind of changes in the behavior and/ or emotional status of [patient's name] did you observe? Open response.

- Would you say that, because of the illness, the life of [patient's name] has been affected in some of the following aspects? Alternatives:
1. Less time to play and interact with other children.
2. More difficulties in meeting with friends
3. Give up caring for a pet
4. Less time/more difficulties for hobbies or favorite sport

5. Difficulties to assist to extra-curricular school activities.
6. Other [Specify]

- Would you say that, because of the illness, the relationship between the child's parents with her/his siblings has been negatively? Response: Yes-No

- Because of the illness, what changes have there been in the type of education received by [patient's name] siblings? For example, change school, start or dropout from kindergarten or nursery. Open response.

- Because of the illness, any members of [patient's name] family (parents and/or siblings), had to relocate to a different city to facilitate treatment? Response: Yes-No

- Related to the negative impact of [patient's name] illness, which of the following alternatives is the best description of the impact of the illness on the family's life? Alternatives:
1. Not affected
2. Slightly affected
3. Moderately affected
4. Severely affected

- Related to the negative impact of [patient's name] illness on the primary caregiver's couple relationship, how much has the illness affected the couple's social life? Alternatives:
1. Not affected
2. Slightly affected
3. Moderately affected
4. Severely affected

- Related to the negative impact of [patient's name] illness on the primary caregiver's couple relationship, how much has the illness affected the couple's intimacy? Alternatives:
1. Not affected
2. Slightly affected
3. Moderately affected
4. Severely affected

- Related to the negative impact of [patient's name] illness on the primary caregiver's couple relationship, how much has the illness affected the couple's communication/dialogue? Alternatives:
1. Not affected
2. Slightly affected
3. Moderately affected
4. Severely affected

- Related to the negative impact of [patient's name] illness on the primary caregiver's couple relationship, how much has the illness affected the couple's emotional attachment? Alternatives:
1. Not affected
2. Slightly affected
3. Moderately affected
4. Severely affected
